# Effects of crude protein and non-essential amino acids on growth performance, blood profile, and intestinal health of weaned piglets

**DOI:** 10.3389/fvets.2023.1243357

**Published:** 2023-11-30

**Authors:** Amanda Medeiros Correia, Jansller Luiz Genova, Alysson Saraiva, Gabriel Cipriano Rocha

**Affiliations:** Muscle Biology and Nutrigenomics Laboratory, Department of Animal Sciences, Universidade Federal de Viçosa, Viçosa, Brazil

**Keywords:** amino acids, crude protein, intestinal health, non-essential amino acids, weaned piglets

## Abstract

This study investigated the effect of crude protein (CP) and non-essential amino acid (NEAA) supplementation on the growth performance, blood profile, intestinal morphology, mRNA relative abundance of inflammatory and antioxidant markers, and tight junction proteins in piglets over the first 2 weeks after weaning. Ninety 21-day-old piglets (7.55 ± 0.72 kg) were assigned in a randomized block design to one of three dietary treatments: (1) high CP, a diet with 24% CP; (2) low CP, a diet with 18% CP; and (3) low CP + NEAA, a diet with 18% CP supplemented with 5 g/kg Arg (L-arginine; purity >99%) and 10 g/kg Glu + Gln (minimum 10% L-glutamine and minimum 10% L-glutamate). Piglets were fed with corn-soybean meal basal diets in a 14-day trial. There was an improvement (*p* < 0.05) in the feed conversion ratio of piglets fed the high-CP diet compared to treatments with low CP or low CP + NEAA. Serum urea nitrogen was higher (*p* < 0.05) in piglets fed high CP compared to other dietary treatments. In the duodenum, the villus height of animals fed the low-CP + NEAA diets was greater (*p* < 0.05) than those fed with the high- and low-CP diets. The goblet cell proportion of piglets fed low CP + NEAA or high CP was higher (*p* < 0.05) compared to low CP. In the jejunum, the crypt depth of the piglets with the high-CP dietary treatment was greater (*p* < 0.05) in comparison with low CP + NEAA. In the jejunum, IFN-γ mRNA expression was higher (*p* < 0.05) in animals fed the high-CP diets compared to other dietary treatments. However, superoxide dismutase and occludin mRNA expression were higher (*p* < 0.05) in animals fed low CP + NEAA than in piglets on the high-CP diets. In the ileum, the number of Peyer’s patches in piglets fed high CP was higher (*p* < 0.05) compared to other dietary treatments. In conclusion, the high-CP diet (24% CP) improves the feed conversion of piglets in the first 2 weeks after weaning compared to the low-CP diet (18% CP) supplemented or not with NEAA. However, the low-CP diet supplemented with NEAA (Arg, Gln, and Glu) improves intestinal health in piglets by promoting greater villus height and proportion of goblet cells in the duodenum, reducing jejunal crypt depth, and reducing Peyer’s number patches in the ileum. In addition, piglets that received the low-CP + NEAA diet showed an increase in superoxide dismutase and occludin and a lower expression of IFN-γ mRNA.

## 1 Introduction

The gastrointestinal tract of the piglet undergoes several changes during the post-weaning period until it is able to digest plant-based feed ingredients. Therefore, it is crucial for the gastrointestinal tract to regulate the changes caused by the introduction of a solid diet, such as gastric-intestinal pH regulation, enzymatic secretion, and intestinal motility, with the aim of improving digestion and nutrient absorption processes.

Soybean meal (SBM) is the most widely used plant protein source for weaned piglets’ diets. The amino acid (AA) profile, balance, and digestibility of SBM are better than any other plant protein source used in swine diets. However, growth performance, intestinal morphology, and immunological status of weaned piglets may be negatively affected due to the presence of antinutritional compounds in this ingredient (1, 2). The reduction of dietary crude protein (CP) coupled with supplementation of industrial AA classified as nutritionally essential (EAA) and non-essential (NEAA) are alternatives to reduce the impacts reported in the post-weaning phase (3–5).

EAA cannot be synthesized by pigs from materials ordinarily available in cells at a rate matching the demands for maintenance, growth, development, and health, which must be provided in the diet to meet the requirements (6). In contrast, NEAAs are AAs that can be synthesized in adequate amounts by the animal organism to meet the requirements for maintenance, growth, development, and health and, therefore, do not need to be provided in the diet (7). During stress, such as health challenges, the synthesis of adequate amounts of NEAAs can be limited by the availability of appropriate amounts of metabolic nitrogen (N) (8). However, NEAAs have a physiological function, and thus the animal may have, in some specific conditions, dietary requirements for NEAAs to support their maximal growth and health (2). Because of an incomplete understanding of AA biochemistry, nutrition, and physiology, the concept of “nutritional non-essentiality” has led to a disregard for the importance of NEAAs in the practice of nutrition (9), resulting in reduced growth performance (5).

Among the NEAAs, arginine (Arg) (10), glutamine (Gln) (11), and glutamate (Glu) (12) can improve the intestinal health of weaned piglets by reducing inflammation and improving the integrity of the intestinal epithelial mucosa. Wu et al. (13) suggested that the effects of Arg are mediated by nitric oxide production and regulation of gene expression related to cell proliferation and differentiation in the intestinal mucosa. Glutamine is the main source of energy for enterocytes, and it is important to maintain the structural and functional integrity of the intestinal mucosa (14). Similarly, Glu is related to increasing the rate of cell proliferation and differentiation and reducing the oxidative stress of intestinal cells by increasing glutathione synthesis (12).

On the other hand, studies have also shown that higher levels of CP in diets for piglets can be beneficial due to the greater contribution of NEAA, peptides, and total N (15–17). According to these authors, CP levels as high as 24% would not compromise piglets’ growth performance, although they could reduce gut health. Moreover, according to Rocha et al. (2), there is a minimum CP level after which the growth performance of pigs can be compromised. For weaned piglets, the proposed minimum CP level was 18.4%. Apparently, below this minimum level, other nutrients such as NEAAs, bioactive compounds, and others become limiting for maximal growth performance.

Based on this knowledge, the hypothesis of this study is that supplementation with NEAAs in low-CP diets can improve the performance, intestinal health, and immune response of weaned piglets. Thus, the study investigated the effect of CP and NEAA supplementation on the growth performance, blood profile, intestinal morphology, mRNA relative abundance of inflammatory and antioxidant markers, and tight junction proteins in piglets over the first 2 weeks after weaning.

## 2 Materials and methods

### 2.1 Animals and housing

Ninety piglets [PIC 337 (Large White × Landrace × Duroc × Pietrain) × Camborough (Large White × Landrace)], castrated male and female, weaned at 21 days old and with 7.55 ± 0.72 kg body weight (BW), were used over the first 2 weeks after weaning. Piglets were housed in suspended pens (0.54 m^2^/piglet) at an experimental facility at the Universidade Federal de Viçosa, MG, Brazil. Each pen houses three piglets with free access to feed and water. For increased microbial pressure, piglets were raised in rooms that were not disinfected or cleaned after the previous occupation by piglets from the same herd (18, 19). This procedure was adopted to simulate the commercial condition of a production unit. The minimum and maximum temperatures inside the nursery room were 27.4 ± 0.7°C and 30.9 ± 0.8°C, respectively.

### 2.2 Diets and experimental design

Diets were formulated according to the nutritional recommendations of the Brazilian Tables for Poultry and Swine (20) (Table 1) and provided in mash form. At 21 days, piglets were assigned in a randomized block design based on BW to one of three dietary treatments: (1) high CP, a diet with 24% CP; (2) low CP, a diet with 18% CP; and (3) low CP + NEAA, a diet with 18% CP supplemented with 5 g/kg Arg (L-arginine; purity >99%) and 10 g/kg Glu + Gln (minimum 10% L-glutamine and minimum 10% L-glutamate). There were 10 pen replicates for each of the three dietary treatments.

**Table 1:** Ingredients and calculated nutritional composition of diets fed to weaned piglets (g/kg, as-fed basis).^a^

Ingredients, g/kg	High CP	Low CP	Low CP + NEAA
Corn, 7.8% CP	318.5	495.5	495.4
Soybean meal, 46.0% CP	261.5	73.5	73.5
Dried whey, 12.5% CP	150.0	150.0	150.0
Soybean micronized, 36.0% CP	100.0	100.0	100.0
Extrude corn, 7.6% CP	55.0	55.0	55.0
Plasma protein, 78.0% CP	40.0	40.0	40.0
Sugar	30.0	30.0	30.0
Dicalcium phosphate	11.7	13.3	13.3
Limestone	8.5	9.1	9.1
Soybean oil	11.0	3.0	3.0
Anti-caking^b^	3.0	3.0	3.0
Zinc oxide	2.5	2.5	2.5
Choline chloride	2.0	2.0	2.0
L-lys, 78.0%	1.3	7.0	7.0
DL-met, 99.0%	1.4	3.1	3.1
L-thr, 98.5%	1.1	3.8	3.8
L-trp, 99.0%	–	1.0	1.0
L-val, 96.5%	–	2.6	2.6
L-ile, 98.0%	–	1.8	1.8
L-leu, 99.5%	–	0.6	0.6
L-his, 98.0%	–	0.7	0.7
L-arg, 98.0%	–	–	5.0
Gln + Glu, 98.0%	–	–	10.0
Salt	0.4	0.4	0.4
Copper sulfate	0.6	0.6	0.6
Vitamin–mineral premix	1.4	1.4	1.4
**Calculated and analyzed** ^**c**^ **composition**			
Metabolizable energy, kcal/kg	3,400	3,400	3,453
Crude protein, %	24.0 (23.4)	18.0	19.6 (19.5)
SID^d^ lys, %	1.45 (1.52)	1.45	1.45 (1.51)
SID met, %	0.43 (0.48)	0.52	0.52 (0.52)
SID met + cys, %	0.81 (0.84)	0.81	0.81 (0.83)
SID thr, %	0.97 (1.11)	0.97	0.97 (1.05)
SID trp, %	0.27 (0.29)	0.27	0.27 (0.28)
SID val, %	1.06 (1.29)	1.00	1.00 (1.19)
SID ile, %	0.94 (0.96)	0.79	0.79 (0.83)
SID leu, %	1.84 (1.93)	1.45	1.45 (1.55)
SID his, %	0.59 (0.57)	0.47	0.47 (0.50)
SID arg, %	1.44 (1.30)	0.90	1.37 (1.22)
Total calcium, %	0.85	0.85	0.85
Available P, %	0.50	0.50	0.50
Sodium, %	0.28	0.28	0.28
Lactose, %	11.2	11.2	11.2

a Dietary treatment: high CP, diet with 24% CP; low CP, diet with 18% CP; low CP + NEAA, diet with 18% CP supplemented with 5 g/kg Arg (L-arginine, purity > 99%) and 10 g/kg Glu + Gln (minimum 10% L-glutamine with minimum 10% L-glutamate).

b Tixosil^®^(Solvay, Brazil) prevents the formation of lumps (caking).

c Total amino acids analyzed are included in the parenthesis.

d Standardized ileal is digestible.

### 2.3 Performance and diarrhea incidence

Throughout the trial, feed was weighed before feeding, and feed wastage was collected and weighed daily to determine the average daily feed intake (ADFI). At 21 and 35 days, piglets were individually weighed to estimate BW, average daily weight gain (ADG), and feed conversion ratio (FC). In addition, diarrhea incidence was visually assessed by the same technician at 7:00 h when piglets were 25, 27, 29, 31, and 33 days of age and were classified as 0 = absence or 1 = presence for each pen (5).

### 2.4 Sample collection

At 35 days of age, blood was collected from one piglet whose BW was closest to the average weight of the piglets within its respective pen. Blood was collected by orbital sinus puncture with a hypodermic needle (40 × 1.6 mm) into 10 mL tubes without anticoagulants for the determination of serum urea N (SUN; Ureal Cobas C311, Linklab, software PNCQ) and immunoglobulin G concentrations (IgG Atellica CH IgG_2, CH Analyzer, Siemens Healthineers). In addition, blood samples were collected in 10 mL tubes containing sodium heparin Now the whole sentence is: In addition, blood samples were collected in 10 mL tubes containing sodium heparin and sent to commercial laboratory (Viçosa Lab, Viçosa-MG, Brazil) to assess the plasma amino acid profile using liquid chromatography–tandem mass spectrometry. to assess the plasma amino acid profile using liquid chromatography–tandem mass spectrometry.

The same blood donor piglet was electrically stunned, followed by exsanguination to collect samples. Fragments measuring 2 cm were sampled from the duodenum (10 cm from the pylorus), jejunum (mid-section), and ileum (5 cm to the ileocecal junction) for histological evaluation (21). The histological sections were then washed in a physiological solution and fixed in 4.0% paraformaldehyde solution for 24 h at room temperature. Another 2 cm of jejunum was collected, immediately frozen in liquid nitrogen, and stored at −80°C for RNA extraction and gene expression analysis.

### 2.5 Intestinal morphology, Peyer’s patches, and goblet cells

After 24 h of fixation, the tissues of the duodenum, jejunum, and ileum were transferred to a 70% (v/v) ethanol solution. Next, they were cross-sectionally cut and dried in ethyl crescent gradients, diaphanized in HistoChoice^®^, and embedded in liquid Paraplast^®^ at 65°C. Five transverse cuts with 5 μm thickness each were placed per slide and stained with hematoxylin and eosin. The cuts were semi-serial, using 1 in 10 cuts. For morphological readings of villus height and crypt depth in the duodenum, jejunum, and ileum, an EVOS M5000 Imaging System (Invitrogen, Thermo Fisher Scientific) optical microscope with a 10-objective lens was used. Afterward, the images were analyzed using the image analyzer ImageJ 1.50i; java1.6.0_20 (National Institutes of Health). The heights of 20 villi and their 20 crypts were selected and measured. Villus:crypt ratios using the length data were then calculated. All measurements were made by a single individual. In the ileum segment, the total count of Peyer’s patches was performed with a magnification of 4 × .

For evaluation of goblet cells in the duodenum, jejunum, and ileum, 10 fields per slide were photographed at a magnification of 20×. Subsequently, the Image J program was used, and perpendicular lines were inserted with markings in uniformly sized quadrants under each image. Then, the total count of intersections in the image and of the cells that touched the intersections was performed. The calculation followed the methodology proposed by Mandarim-de-Lacerda (22):


$$\mathrm{Goblet}\;\mathrm{cells}\;\left(\%\right)=\frac{\mathrm{total}\;\mathrm{number}\;\mathrm{of}\;\mathrm{goblet}\;\mathrm{cells}\times 100}{\mathrm{total}\;\mathrm{number}\;\mathrm{of}\;\mathrm{intersections}\text{.}}$$

### 2.6 Relative mRNA abundance

Total RNA extraction was performed using a commercial kit (SV Total RNA isolation kit—Promega, Z3100) following the manufacturer’s instructions. The RNA concentration was estimated using NanoDrop™ Lite (Thermo Fisher Scientific), and RNA integrity was evaluated through 1% agarose gel electrophoresis. Complementary DNA synthesis was performed according to the GoScript™ Reverse Transcription System protocol (Promega Corporation). GenBank numbers to access the primers for the genes are shown in Table 2. Primers were used for reverse transcription quantitative PCR with GoTaq^®^ qPCR Master Mix (Promega) in QuantStudio^®^ 3 (Applied Biosystems, Thermo Fisher Scientific). Geometric mean of the Ct value of β-actin was used to normalize target gene expression in the jejunum samples. Gene of interest relative expression was calculated by 2^−ΔΔCt^ (23) for glutathione peroxidase (*GPX*), superoxide dismutase (*SOD*), catalase (*CAT*), occludin (*OCL*), zonula occludens-1 (*ZO-1*), interferon gamma (*IFN-γ*), tumor necrosis factor-alpha (*TNF-α*), interleukin 1 beta (*IL1-β*), and interleukin 10 (*IL-10*).

**Table 2:** List of primers used in reverse transcription quantitative-PCR gene expression analysis in weaned piglets.

Genes^a^	GenBank number	Sequence^b^
*GPX*	NM_214201.1	F: 5′-GCCCAACTTCATGCTCTTC-3′
		R: 5′-CAGGATCTCCCCATTCTTGGC-3′
*SOD*	NM_001190422.1	F: 5′-ATCAAGAGAGGCACGTTGGA-3′
		R: 5′-TCTGCCCAAGTCATCTGGTT-3′
*CAT*	NM_214301.2	F: 5′-GCTTTAGTGCTCCCGAACAG-3′
		R: 5′-AGATGACCCGCAATGTTCTC-3′
*OCL*	NM_001163647.1	F: 5′-TCCTGGGTGTGATGGTGTTC-3′
		R: 5′-CGTAGAGTCCAGTCACCGCA-3′
*ZO-1*	XM_003353439.2	F: 5′-AAGCCCTAAGTTCAATCACAATCT-3′
		R: 5′-ATCAAACTCAGGAGGCGGC-3′
*IFN-γ*	NM_213948	F: 5′-TGGTAGCTCTGGGAAACTGAATG-3′
		R: 5′-GGCTTTGCGCTGGATCTG-3′
*TNF-α*	NM_214022.1	F: 5′-CATCGCCGTCTCCTACCA-3′
		R: 5′-CCCAGATTCAGCAAAGTCCA-3′
*IL1-β*	NM_214055.1	F: 5′-TCTGCCCTGTACCCCAACTG-3′
		R: 5′-CCCAGGAAGACGGGCTTT-3′
*IL-10*	NM_214041.1	F: 5′-GAAGGACCAGATGGGCGACTT-3′
		R: 5′-CACCTCCTCCACGGCCCTTG-3′
β-actin	U07786.1	F: 5′-CTCTTCCATCGTGTCCTTCTAC-3′
		R: 5′-CCTCAGACTTGTCGATCTTCTG-3′

a *GPX*, glutathione peroxidase; *SOD*, superoxide dismutase; *CAT*, catalase; *OCL*, occludin; *ZO-1*, zonula occludens-1; *IFN-γ*, interferon gamma; *TNF-α*, tumor necrosis factor alpha; *IL1-β*, interleukin 1 beta; *IL-10*, interleukin 10.

b F and R indicate forward and reverse primers, respectively.

### 2.7 Statistical analysis

The pen was considered the experimental unit for growth performance and diarrhea incidence analysis. One piglet from each pen was considered the experimental unit for intestinal morphology, gene expression, and serum results. The statistical model included the fixed effect of treatment, and block and residual errors as random factors. The normality of experimental errors was evaluated using the Shapiro–Wilk test. The data were analyzed using the GLMMMIX procedure of SAS 9.4 (SAS Inst., Inc., Cary, NC, United States) via one-way analysis of variance (ANOVA). When an effect was detected in the ANOVA (*p* < 0.05), means were compared using Tukey’s *post-hoc* test. Data on diarrhea were analyzed using the FREQ procedure of SAS, and the effects were determined using the chi-squared test at *p* < 0.05.

## 3 Results

### 3.1 Growth performance and fecal consistency score

There was no effect (*p* > 0.05) of dietary treatments on ADFI, ADG, and final BW (Table 3). However, there was an improvement (*p* < 0.05) in the FC of piglets fed the high-CP diet compared to treatments with low CP or low CP + NEAA. Treatments did not alter (*p* > 0.05) the diarrhea incidence (Table 4).

**Table 3:** Effects of crude protein and non-essential amino acids on growth performance of piglets (at 35 days old).^1^

Item^2^	Dietary treatment^3^			SEM^4^	*p*-value
	High CP	Low CP	Low CP + NEAA		
Initial BW, kg	7.56	7.56	7.55	–	–
ADFI, g/day	399	395	424	22.28	0.78
ADG, g/day	345	298	333	19.32	0.31
FC, g:g	1.16^b^	1.33^a^	1.27^a^	0.02	<0.01
Final BW, kg	12.37	11.75	12.22	0.31	0.47

^a,b^Means with different superscript letters are different by Tukey’s *post-hoc* test at 5% probability.

1 Data are means of 10 pens replicated per dietary treatment and 3 piglets per pen as an experimental unit.

2 Average daily feed intake (ADFI, g/day), average daily weight gain (ADG, g/day), feed conversion ratio (FC).

3 Dietary treatment: high CP, diet with 24% CP; low CP, diet with 18% CP; low CP + NEAA, diet with 18% CP supplemented with 5 g/kg Arg (L-arginine, purity > 99%), and 10 g/kg Glu + Gln (minimum 10% L-glutamine with minimum 10% L-glutamate).

4 Pooled standard error of the mean.

**Table 4:** Effects of crude protein and non-essential amino acids on diarrhea incidence of piglets.^a^

Days of age	Dietary treatment^b^			*p*-value
	High CP	Low CP	Low CP + NEAA	
25	0	0	1	0.37
27	2	1	2	0.85
29	1	0	1	0.78
31	2	0	0	0.26
33	2	0	0	0.09

a Data are means of 10 pen replicates per dietary treatment and 3 piglets per pen as an experimental unit.

b Dietary treatment: high CP, diet with 24% CP; low CP, diet with 18% CP; low CP + NEAA, diet with 18% CP supplemented with 5 g/kg Arg (L-arginine, purity > 99%), and 10 g/kg Glu + Gln (minimum 10% L-glutamine with minimum 10% L-glutamate).

### 3.2 Blood profile

The SUN was higher (*p* < 0.05) in piglets fed the high-CP treatment than those with the low-CP and low-CP + NEAA diets (Table 5). There was no effect (*p* > 0.05) of treatments on IgG concentrations. Plasma Gln + Lys concentration was higher (*p* < 0.05) in piglets on high-CP treatment than in low CP, while low CP + NEAA had intermediate results. Plasma Met concentration was higher (*p* < 0.05) in piglets fed low CP than those piglets that received high CP, while low CP + NEAA had intermediate results. Plasma Arg, Orn, and Glu concentrations were higher (*p* < 0.05) in piglets fed high-CP and low-CP + NEAA dietary treatment compared to low CP. Plasma Val concentration was higher in piglets receiving low-CP + NEAA dietary treatment than those with high CP, while low CP had intermediate results. Plasm Leu + Ile, Tyr, and Phe concentrations were higher (*p* < 0.05) in piglets on high-CP dietary treatment compared to others. Plasma Ala concentration was higher (*p* < 0.05) in piglets from low-CP + NEAA treatment compared to others. Plasma Thr, Try, Gly, and Cit concentrations were not influenced (*p* > 0.05) by dietary treatments.

**Table 5:** Effects of crude protein and non-essential amino acids on the blood profile of piglets (at 35 days old).^1^

Item^2^	Dietary treatment^3^			SEM^4^	*p*-value
	High CP	Low CP	Low CP + NEAA		
SUN, mg/dL	21.0^a^	5.7^b^	7.0^b^	0.97	<0.01
IgG, mg/dL	203.3	161.6	170.0	23.09	0.37
**Amino acids,** μ**mol/L**					
Glutamine + lysine	51.2^a^	37.1^b^	44.9^ab^	4.17	0.03
Methionine	39.6^b^	87.4^a^	61.0^ab^	11.61	0.01
Arginine	93.0^a^	40.0^b^	81.1^a^	3.53	<0.01
Threonine	57.2	88.3	79.8	14.24	0.28
Tryptophan	25.2	21.7	24.1	1.74	0.34
Valine	129.3^b^	144.1^ab^	181.6^a^	12.20	0.01
Leucine + isoleucine	146.1^a^	92.2^b^	97.5^b^	5.81	<0.01
Glycine	448.2	410.5	405.3	34.66	0.63
Tyrosine	74.4^a^	30.6^b^	25.2^b^	3.63	<0.01
Ornithine	72.2^a^	39.3^b^	58.1^a^	5.13	<0.01
Phenylalanine	40.0^a^	23.9^b^	16.5^b^	2.58	<0.01
Citrulline	46.4	34.2	36.9	4.32	0.12
Glutamate	146.1^a^	84.5^b^	129.8^a^	8.50	<0.01
Alanine	199.3^b^	173.7^b^	254.5^a^	11.65	<0.01

^a,b^Means with different superscript letters are different by Tukey’s *post-hoc* test at 5% probability.

1 Data are means of 10 piglets per dietary treatment.

2 SUN, serum urea nitrogen; IgG, immunoglobulin G.

3 Dietary treatment: high CP, diet with 24% CP; low CP, diet with 18% CP; low CP + NEAA, diet with 18% CP supplemented with 5 g/kg Arg (L-arginine, purity > 99%) and 10 g/kg Glu + Gln (minimum 10% L-glutamine with minimum 10% L-glutamate).

4 Pooled standard error of the mean.

### 3.3 Intestinal morphology, Peyer’s patches, and goblet cells

In the duodenum, the villus height of animals fed the low-CP + NEAA diets was greater (*p* < 0.05) than those fed with the high- and low-CP diets (Table 6). Moreover, the goblet cell proportion of piglets fed high CP or low CP + NEAA was higher (*p* < 0.05) compared to low CP. However, there were no effects (*p* > 0.05) of treatments on the crypt depth or villus:crypt ratio. In the jejunum, the crypt depth of the piglets with the high-CP dietary treatment was greater (*p* < 0.05) in comparison with low CP + NEAA, while low CP had intermediate results. However, dietary treatments had no effect (*p* > 0.05) on villus height, villus:crypt ratio, or proportion of goblet cells. In the ileum, dietary treatments had no effects (*p* > 0.05) on villus height, crypt depth, villus:crypt ratio, or proportion of goblet cells. However, the number of Peyer’s patches in piglets fed high CP was higher (*p* < 0.05) compared to other dietary treatments.

**Table 6:** Effects of crude protein and non-essential amino acids on the intestinal morphology of piglets (at 35 days old).^1^

Item	Dietary treatment^2^			SEM^3^	*P*-value
	High CP	Low CP	Low CP + NEAA		
**Duodenum**					
Villus height, μm	381^b^	383 ^b^	427 ^a^	11.49	0.01
Crypt depth, μm	208	213	229	8.83	0.40
Villus:crypt ratio	1.8	1.8	1.9	0.07	0.58
Goblet cells, %	53.2^a^	45.3^b^	53.4^a^	2.24	0.01
**Jejunum**					
Villus height, μm	365	324	299	22.00	0.16
Crypt depth, μm	161^a^	143^ab^	141^b^	5.05	0.01
Villus:crypt ratio	2.3	2.2	2.1	0.12	0.67
Goblet cells, %	45.3	44.8	43.6	2.88	0.45
**Ileum**					
Villus height, μm	249	239	244	15.97	0.86
Crypt depth, μm	135	131	132	6.07	0.95
Villus:crypt ratio	1.9	1.9	1.9	0.07	0.90
Goblet cells, %	40.9	44.7	43.0	1.99	0.57
Peyer’s patches, *n*	48^a^	38^b^	41^b^	2.20	0.02

^a,b^Means with different superscript letters are different by Tukey’s *post-hoc* test at 5% probability.

1 Data are means of 10 piglets per dietary treatment.

2 Dietary treatment: high CP, diet with 24% CP; low CP, diet with 18% CP; low CP + NEAA, diet with 18% CP supplemented with 5 g/kg Arg (L-arginine, purity > 99%) and 10 g/kg Glu + Gln (minimum 10% L-glutamine with minimum 10% L-glutamate).

3 Pooled standard error of the mean.

### 3.4 Relative mRNA abundance

**Figure 1:**
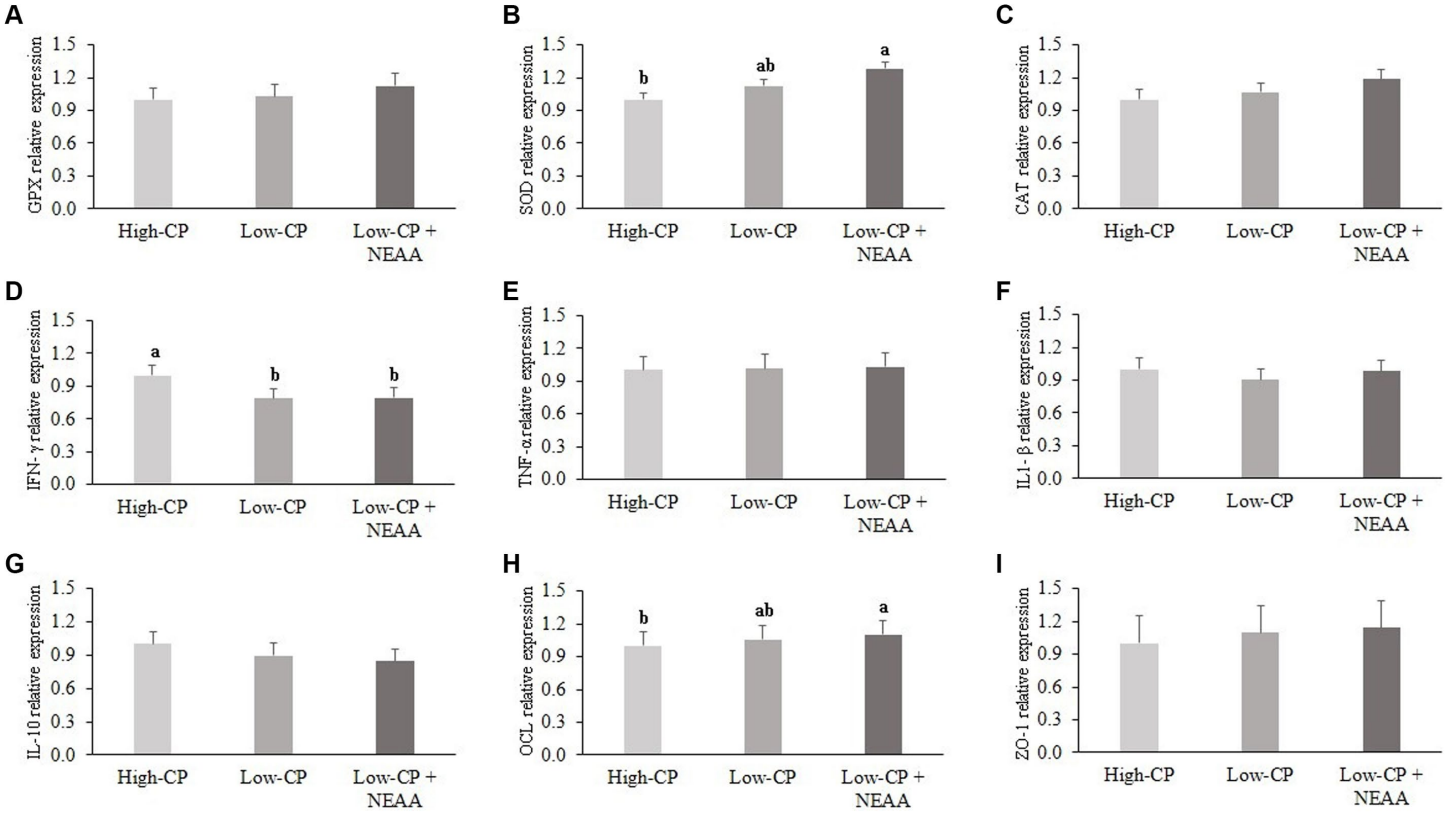
Effects of crude protein and non-essential amino acids on the mRNA relative abundance of inflammatory and antioxidant markers and tight junction proteins of the jejunum of piglets (at 35 days old). Dietary treatments: high CP, diet with 24% CP; low CP, diet with 18% CP; low CP + NEAA, diet supplemented with 5 g/kg Arg (L-arginine; purity >99%), and 10 g/kg Glu + Gln (minimum 10% L-glutamine with minimum 10% L-glutamate). Results are relative to the high-CP treatment. Data are means of 10 piglets per dietary treatment. ^a,b^Means with different superscript letters are different by Tukey’s *post-hoc* test at 5% probability. GPX, glutathione peroxidase **(A)**; SOD, superoxide dismutase **(B)**; CAT, catalase **(C)**; IFN-γ, interferon gamma **(D)**; TNF-α, tumor necrosis factor **(E)**; IL1-β, interleukin 1 beta **(F)**; IL-10, interleukin 10 **(G)**; OCL, occludin **(H)**; ZO-1, zonula occludens-1 **(I)**.

In the jejunum, *IFN-γ* mRNA expression was higher (*p* < 0.05) in animals fed the high-CP diets compared to other dietary treatments (Figure 1). However, *SOD* and *OCL* mRNA expression were higher (*p* < 0.05) in animals fed low CP + NEAA than in piglets on high-CP diets. There was no effect (*p* > 0.05) of dietary treatments on mRNA expression of *GPX*, *CAT*, *TNF-α*, *ZO-1*, *IL1-β*, and *IL-10*.

## 4 Discussion

The reduction of dietary CP balanced with EAA has been used as part of a strategy to improve intestinal health in pigs and, consequently, improve growth performance (4). However, under stress such as the post-weaning period, there is a greater demand for NEAAs because tissue production does not meet the systemic needs (9). Thus, it has been suggested that the generation of NEAAs from EAAs may become a limiting factor for the normal growth performance of weaned pigs (2, 7, 24). In this way, studies have shown that high-CP levels or supplementation of NEAAs in diets for newly weaned piglets may be beneficial due to the higher intake of NEAAs (15–17).

In the present study, three experimental diets were fed to piglets in the first 2 weeks after weaning. The first diet contained 24% CP, supplemented with Lys, Met, and Thr. The second diet contained 18% CP, supplemented with Lys, Met, Thr, Trp, Val, Ile, Leu, and His. The third diet was similar to the second and supplemented with NEAA Arg, Gln, and Glu. All diets were formulated with the EAA at or above the recommended ratio to Lys (20). The hypothesis of the study was that low-CP diets supplemented with NEAAs would improve the growth performance, gut health, and immune response of weaned piglets.

High-CP levels may be associated with a higher incidence of diarrhea (25) and worse growth performance in weaned piglets (26). However, in the present study, the high-CP diets had no negative effects on the incidence of diarrhea, ADG, ADFI, and BW at 35 days of age. In addition, high-CP diets improved the FC of piglets. Others also demonstrated improved growth performance associated with higher levels of dietary CP (27–29). According to Silva et al. (30), reducing dietary CP levels decreases the supply of dietary N and NEAAs, as well as the expression of digestive enzyme genes for carbohydrates and proteases in pigs (31). Therefore, it is assumed that in the present study, inadequate endogenous NEAA synthesis limited the growth of piglets fed the low-CP diets. Moreover, the supplementation of NEAAs in the low-CP + NEAA treatment may not have been sufficient to recover growth performance, probably because the animals required a higher level of NEAAs or other non-supplemented NEAAs. According to Gloaguen et al. (32), the rate of NEAA synthesis can be limited by the availability of dietary or metabolic N, originating from the deamination of EAA, which will further limit the growth performance of animals.

The SUN is indicative of the efficiency of N utilization by the animals. In the present study, piglets fed the low-CP and low-CP + NEAA diets had lower SUN concentrations compared to the high-CP diets. According to Heo et al. (25), AA absorbed beyond what is necessary for biosynthesis cannot be stored and undergoes catabolism, which has urea as its final product. The present result indicated that there was an excess of AA in the high-CP diet. Thus, animals fed the low-CP diets may have been more efficient in N utilization, corroborating the results reported by other authors (5, 16, 17).

Plasma concentrations of AA can be influenced either by the uptake of AA from the diet or by the tissue absorption of circulating AA (33). The lower plasma concentrations of Arg and Glu in piglets fed the low-CP diet may be related to the lower level in the diet and lower availability of N for the synthesis of these AAs as compared to low CP + NEAA and high CP. Piglets fed the high-CP diet had lower concentrations of Met and Val in the plasma, which can be explained by the lower dietary supplementation of these AAs in industrial form. Supplemented industrial AAs are readily available for absorption and are promptly absorbed in the proximal small intestine, while CP-bound AAs need to be broken down by luminal and brush border enzymes before absorption (34, 35). Plasma concentrations of Leu + Ile, Phe, and Tyr were higher in piglets fed the high-CP diet, which is explained by the fact that this diet contained a higher concentration of those AAs as a result of the higher CP content. Moreover, low-CP + NEAA treatment increased plasma concentrations of Orn and Ala compared to low CP, showing that dietary NEAA supplementation may reduce the intestinal catabolism of other AA and elevate their entry into the portal vein, as reported by Yi et al. (3).

Gut health has significant implications for swine health status and nutrient utilization due to its various functions, including digestion and absorption of nutrients, secretion of mucins and immunoglobulins, and selective barrier protection against harmful antigens and pathogens (35). Thus, the evaluation of intestinal morphometry, goblet cells, and Peyer’s patches associated with gene expression of tight junction proteins, pro- and anti-inflammatory cytokines, and antioxidant enzymes can be used as tools for assessing intestinal health (19).

In the present study, animals fed the low-CP + NEAA diet had higher villus height in the duodenum, indicating greater absorptive capacity for the available nutrients (36). This result may be related to the supplementation of Gln and Glu. Glutamine is a major metabolic fuel for rapidly dividing cells, such as enterocytes, and together with Glu, it is related to increasing the rate of cell proliferation and differentiation (12, 14). Piglets fed low-CP + NEAA also had shorter crypt depth in the jejunum, indicating decreased metabolic cost of epithelium turnover associated with inflammation response (35). In addition to shorter crypt depth, those piglets had reduced Peyer’s patches in the ileum, suggesting less intestinal challenge compared to the high-CP diet. Peyer’s patches are aggregated lymphoid follicles, with a protective function against pathogens (37). The high-CP content, as a result of the high SBM level, may have increased the proliferation of pathogenic bacteria in the ileum (although not evaluated in the present study), stimulating the immune system and increasing the number of Peyer’s patches. These results are supported by a study conducted by Deng et al. (1), who reported that the higher the SBM content in the diet, the higher the content of indigestible carbohydrates (stachyose and raffinose) and antigenic proteins (glycinin and β-conglycinin) considered antinutritional factors. In addition, the high-CP content can increase the proliferation of pathogenic bacteria and their potential toxins for the gastrointestinal tract, such as ammonia and polyamines (38).

Goblet cells are responsible for the production of mucus that acts as a physical barrier against the invasion of pathogens, while tight junction proteins form a selective physical barrier to prevent endotoxin absorption (39). In the present study, it was demonstrated higher proportion of goblet cells and higher expression of OCLN in animals fed low-CP + NEAA diets, thus indicating improved intestinal integrity. Additionally, the expression of antioxidant enzymes and cytokines in the jejunum of weaned piglets was evaluated because the antioxidant capacity and the immune response are fundamental for the promotion of intestinal health. According to Yin et al. (40), weaning causes an increase in reactive oxygen species that can cause oxidative stress at the intestinal level and in other tissues. In this way, it has been shown that supplementation of Arg, Gln, and Glu in diets can improve the intestinal antioxidant response in pigs (3, 41). Corroborating this report, animals fed a low-CP + NEAA diet showed increased SOD expression in the jejunum, which suggested greater antioxidant capacity associated with NEAA supplementation.

IFN-γ is a pro-inflammatory cytokine considered an immunological marker produced in response to inflammation (42). Animals fed the high-CP diet had higher expression of IFN-γ, which may be related to higher SBM levels compared to the low-CP treatments (261 × 73 g/kg). High levels of indigestible proteins in the diet might result in inflammatory response, especially by increasing pro-inflammatory cytokine levels, which might decrease gut integrity (43). Actually, in the present study, the higher IFN-γ in pigs fed high-CP diets was associated with reduced OCL expression.

Altogether, the results indicated that the low-CP + NEAA diet improves N utilization efficiency and intestinal architecture and modulates the response expression of genes related to the immune system and antioxidant capacity in piglets in the first 2 weeks after weaning. Therefore, supplementation with Arg, Gln, and Glu in diets for weaned piglets is a promising nutritional approach to support a formulation with low dietary CP levels.

## 5 Conclusion

The high-CP diet (24% CP) improves the feed conversion of piglets in the first 2 weeks after weaning compared to the low-CP diet (18% CP) supplemented or not with NEAAs. However, the low-CP diet supplemented with NEAA (5 g/kg of Arg and 10 g/kg of Gln + Glu) improves intestinal health in piglets by promoting greater villus height and proportion of goblet cells in the duodenum, reducing jejunal crypt depth, and reducing Peyer’s number patches in the ileum. In addition, piglets that received the low-CP + NEAA diet showed an increase in *SOD* and *OCL* mRNA expression and a lower expression of *IFN-γ* mRNA.

## Data availability statement

The original contributions presented in the study are included in the article/supplementary materials, further inquiries can be directed to the corresponding author.

## Ethics statement

The animal study was approved by Ethical Committee on Animal Use of Universidade Federal de Viçosa (UFV) protocol n° 066/2021. The study was conducted in accordance with the local legislation and institutional requirements.

## Author contributions

AC, AS, and GR: conceptualization, design of the study, and writing—original draft preparation. AC: carrying out the project. AC, JG, and GR: methodology, statistical analysis, formal analysis, and writing—review and editing. All authors contributed to the article and approved the submitted version.

## References

[1] Deng Z Duarte ME Jang KB Kim SW. Soy protein concentrate replacing animal protein supplements and its impacts on intestinal immune status, intestinal oxidative stress status, nutrient digestibility, mucosa-associated microbiota, and growth performance of nursery pigs. J Anim Sci. (2022) 100:skac255. doi: 10.1093/jas/skac255, PMID: 35950990 PMC9576021

[2] Rocha GC Duarte ME Kim SW. Advances, implications, and limitations of low-crude-protein diets in pig production. Animals. (2022) 12:3478. doi: 10.3390/ani12243478, PMID: 36552397 PMC9774321

[3] Yi D Li B Hou Y Wang L Zhao D Chen H. Dietary supplementation with an amino acid blend enhances intestinal function in piglets. Amino Acids. (2018) 50:1089–100. doi: 10.1007/s00726-018-2586-7, PMID: 29770867

[4] Zhao Y Weaver AC Fellner V Payne RL Kim SW. Amino acid fortified diets for weanling pigs replacing fish meal and whey protein concentrate: effects on growth, immune status, and gut health. J Anim Sci Biotechnol. (2014) 5:1–10. doi: 10.1186/2049-1891-5-57, PMID: 25838896 PMC4383190

[5] Gomes MS Júnior DTV Silva FCO Júnior RLC Junior VR Saraiva A. Effects of glutamine and glutamate on nursey piglets fed diets with different digestible lysine content. Semin Ciênc Agrár. (2021) 42:3919–30. doi: 10.5433/1679-0359.2021v42n6SUPL2p3919

[6] National Research Council. Nutrient requirements of swine. Washington, DC: The National Academies Press (2012).

[7] Wu G. Functional amino acids in growth, reproduction, and health. Adv Nutr. (2010) 1:31–7. doi: 10.3945/an.110.1008, PMID: 22043449 PMC3042786

[8] Rochell SJ Alexander LS Rocha GC Van Alstine WG Boyd RD Pettigrew JE. Effects of dietary soybean meal concentration on growth and immune response of pigs infected with porcine reproductive and respiratory syndrome virus. J Anim Sci. (2015) 93:2987–97. doi: 10.2527/jas.2014-8462, PMID: 26115285

[9] Hou Y Yin Y Wu G. Dietary essentiality of “nutritionally non-essential amino acids” for animals and humans. Exp Biol Med. (2015) 240:997–1007. doi: 10.1177/1535370215587913, PMID: 26041391 PMC4935284

[10] Liao SF. Invited review: maintain or improve piglet gut health around weanling: the fundamental effects of dietary amino acids. Animals. (2021) 11:1110. doi: 10.3390/ani11041110, PMID: 33924356 PMC8069201

[11] He J Feng GD Ao X Li YF Qian HX Liu JB. Effects of L-glutamine on growth performance, antioxidant ability, immunity and expression of genes related to intestinal health in weanling pigs. Livest Sci. (2016) 189:102–9. doi: 10.1016/j.livsci.2016.05.009

[12] Yin J Liu M Ren W Duan J Yang G Zhao Y. Effects of dietary supplementation with glutamate and aspartate on diquat-induced oxidative stress in piglets. PLoS One. (2015) 10:e0122893. doi: 10.1371/journal.pone.0122893, PMID: 25875335 PMC4398417

[13] Wu G Bazer FW Dai Z Li D Wang J Wu Z. Amino acid nutrition in animals: protein synthesis and beyond. Annu Rev Anim Biosci. (2014) 2:387–417. doi: 10.1146/annurev-animal-022513-114113 25384149

[14] Ji FJ Wang LX Yang HS Hu A Yin YL. The roles and functions of glutamine on intestinal health and performance of weaning pigs. Animal. (2019) 13:2727–35. doi: 10.1017/S1751731119001800, PMID: 31407650

[15] Batson KL Calderón HI Tokach MD Woodworth JC Goodband RD Dritz SS. Effects of feeding diets containing low crude protein and coarse wheat bran as alternatives to zinc oxide in nursery pig diets. J Anim Sci. (2021) 99:skab090. doi: 10.1093/jas/skab090, PMID: 33755175 PMC8269968

[16] Nyachoti CM Omogbenigun FO Rademacher M Blank G. Performance responses and indicators of gastrointestinal health in early-weaned pigs fed low-protein amino acid-supplemented diets. J Anim Sci. (2006) 84:125–34. doi: 10.2527/2006.841125x, PMID: 16361499

[17] Yue LY Qiao SY. Effects of low-protein diets supplemented with crystalline amino acids on performance and intestinal development in piglets over the first 2 weeks after weaning. Livest Sci. (2008) 115:144–52. doi: 10.1016/j.livsci.2007.06.018

[18] Le Floc'h N Jondreville C Matte JJ Seve B. Importance of sanitary environment for growth performance and plasma nutrient homeostasis during the post-weaning period in piglets. Arch Anim Nutr. (2006) 60:23–34. doi: 10.1080/17450390500467810, PMID: 16529155

[19] Valini GAC Duarte MS Calderano AA Teixeira LM Rodrigues GA Fernandes KM. Dietary nucleotide supplementation as an alternative to in-feed antibiotics in weaned piglets. Animal. (2021) 15:100021. doi: 10.1016/j.animal.2020.100021, PMID: 33573936

[20] Rostagno HS Albino LFT Hannas MI Donzele JL Sakomura NK Perazzo FG. Brazilian tables for poultry and swine: composition of feedstuffs and nutritional requirements. Viçosa: Universidade Federal de Viçosa (2017).

[21] Yang KM Jiang ZY Zheng CT Wang L Yang XF. Effect of *Lactobacillus plantarum* on diarrhea and intestinal barrier function of young piglets challenged with enterotoxigenic *Escherichia coli* K88. J Anim Sci. (2014) 92:1496–503. doi: 10.2527/jas.2013-6619, PMID: 24492550

[22] Mandarim-de-Lacerda CA. Métodos quantitativos em morfologia. Rio de Janeiro: Eduerj (1995).

[23] Livak KJ Schmittgen TD. Analysis of relative gene expression data using real-time quantitative PCR and the 2^− ΔΔCT^ method. Methods. (2001) 25:402–8. doi: 10.1006/meth.2001.1262 11846609

[24] Fukatsu K Kudsk KA. Nutrition and gut immunity. Surg Clin North Am. (2011) 91:755–70. doi: 10.1016/j.suc.2011.04.007, PMID: 21787966 PMC3144400

[25] Heo JM Kim JC Hansen CF Mullan BP Hampson DJ Pluske JR. Feeding a diet with decreased protein content reduces indices of protein fermentation and the incidence of postweaning diarrhea in weaned pigs challenged with an enterotoxigenic strain of *Escherichia coli*. J Anim Sci. (2009) 87:2833–43. doi: 10.2527/jas.2008-1274, PMID: 19502498

[26] Wu G. Dietary requirements of synthesizable amino acids by animals: a paradigm shift in protein nutrition. J Anim Sci Biotechnol. (2014) 5:1–12. doi: 10.1186/2049-1891-5-34, PMID: 24999386 PMC4082180

[27] Kim JC Heo JM Mullan BP Pluske JR. Efficacy of a reduced protein diet on clinical expression of post-weaning diarrhoea and life-time performance after experimental challenge with an enterotoxigenic strain of *Escherichia coli*. Anim Feed Sci Technol. (2011) 170:222–30. doi: 10.1016/j.anifeedsci.2011.08.012

[28] Limbach JR Espinosa CD Perez-Calvo E Stein HH. Effect of dietary crude protein level on growth performance, blood characteristics, and indicators of intestinal health in weanling pigs. J Anim Sci. (2021) 99:skab166. doi: 10.1093/jas/skab166 34019637 PMC8202089

[29] Tian ZM Ma XY Yang XF Fan QL Xiong YX Qiu YQ. Influence of low protein diets on gene expression of digestive enzymes and hormone secretion in the gastrointestinal tract of young weaned piglets. J Zhejiang Univ Sci B. (2016) 17:742–51. doi: 10.1631/jzus.B1600229, PMID: 27704744 PMC5064168

[30] Silva KE Mansilla WD Shoveller AK Htoo JK Cant JP de Lange CF. The effect of supplementing glycine and serine to a low crude protein diet on growth and skin collagen abundance of nursery pigs. J Anim Sci. (2020) 98:skaa023. doi: 10.1093/jas/skaa023, PMID: 31965147 PMC7021636

[31] He L Wu L Xu Z Li T Yao K Cui Z. Low-protein diets affect ileal amino acid digestibility and gene expression of digestive enzymes in growing and finishing pigs. Amino Acids. (2016) 48:21–30. doi: 10.1007/s00726-015-2059-1, PMID: 26210756

[32] Gloaguen M Le Floc'H N Corrent E Primot Y Van Milgen J. The use of free amino acids allows formulating very low crude protein diets for piglets. J Anim Sci. (2014) 92:637–44. doi: 10.2527/jas.2013-6514, PMID: 24398840

[33] Ren M Zhang SH Zeng XF Liu H Qiao SY. Branched-chain amino acids are beneficial to maintain growth performance and intestinal immune-related function in weaned piglets fed protein restricted diet. Asian-Aust J Anim Sci. (2015) 28:1742–50. doi: 10.5713/ajas.14.0131, PMID: 26580442 PMC4647083

[34] Morales A Buenabad L Castillo G Vázquez L Espinoza S Htoo JK. Dietary levels of protein and free amino acids affect pancreatic proteases activities, amino acids transporters expression and serum amino acid concentrations in starter pigs. J Anim Physiol Anim Nutr. (2017) 101:723–32. doi: 10.1111/jpn.12515, PMID: 27121753

[35] Yang Z Liao SF. Physiological effects of dietary amino acids on gut health and functions of swine. Front Vet Sci. (2019) 6:169. doi: 10.3389/fvets.2019.00169, PMID: 31245390 PMC6579841

[36] Zhang S Qiao S Ren M Zeng X Ma X Wu Z. Supplementation with branched-chain amino acids to a low-protein diet regulates intestinal expression of amino acid and peptide transporters in weanling pigs. Amino Acids. (2013) 45:1191–205. doi: 10.1007/s00726-013-1577-y, PMID: 23990159

[37] Mair KH Sedlak C Käser T Pasternak A Levast B Gerner W. The porcine innate immune system: an update. Dev Comp Immunol. (2014) 45:321–43. doi: 10.1016/j.dci.2014.03.022, PMID: 24709051 PMC7103209

[38] Duarte ME Kim SW. Intestinal microbiota and its interaction to intestinal health in nursery pigs. Anim Nutr. (2022) 8:169–84. doi: 10.1016/j.aninu.2021.05.001, PMID: 34977387 PMC8683651

[39] Moeser AJ Pohl CS Rajput M. Weaning stress and gastrointestinal barrier development: implications for lifelong gut health in pigs. Anim Nutr. (2017) 3:313–21. doi: 10.1016/j.aninu.2017.06.003, PMID: 29767141 PMC5941262

[40] Yin J Wu MM Xiao H Ren WK Duan JL Yang G. Development of an antioxidant system after early weaning in piglets. J Anim Sci. (2014) 92:612–9. doi: 10.2527/jas.2013-6986, PMID: 24352957

[41] Jiao N Wu Z Ji Y Wang B Dai Z Wu G. L-glutamate enhances barrier and antioxidative functions in intestinal porcine epithelial cells. J Nutr. (2015) 145:2258–64. doi: 10.3945/jn.115.217661, PMID: 26338884

[42] Andrade MER Araújo RS de Barros PAV Soares ADN Abrante FA Generoso SV. The role of immunomodulators on intestinal barrier homeostasis in experimental models. Clin Nutr. (2015) 34:1080–7. doi: 10.1016/j.clnu.2015.01.012, PMID: 25660317

[43] Long S Ma J Piao X Li Y Rasmussen SH Liu L. Enzyme-treated soybean meal enhanced performance via improving immune response, intestinal morphology and barrier function of nursery pigs in antibiotic free diets. Animals. (2021) 11:2600. doi: 10.3390/ani11092600, PMID: 34573566 PMC8471553

